# Evaluating strategies used by family caregivers in response to daily behavioral symptoms of dementia.

**DOI:** 10.1093/geroni/igaf122.2044

**Published:** 2025-12-31

**Authors:** Wesley Browning

**Affiliations:** UTHealth Houston Cizik School of Nursing, Houston, Texas, United States

## Abstract

Alzheimer’s disease and related dementias (ADRD) are marked by behavioral symptoms (BSD) that often complicate their care by family members. These symptoms include care refusals, delusions, mood difficulties, along with inappropriate, compulsive, and safety-related behaviors (i.e., property damage, aggression, and suicidality). These behaviors are frequently associated with caregiving behaviors, including elder mistreatment, and ultimately, worse caregiving outcomes. Potentially inappropriate medication (PIM) use is one form of mistreatment that has been studied in long-term care settings, but has received little attention in family caregiving research. This study (N = 85, observations=2012) evaluated descriptive data from an ongoing study investigating daily caregiving strategies, including pharmacological and nonpharmalogical strategies, to determine what strategies were most commonly used in response to BSDs. Caregivers frequently reported using nonpharmacological strategies (70-91%) to manage BSDs; however, medications were frequently used to stop safety-related (19.9%), aggressive (27.2%), and inappropriate (29.6%) behaviors or make them easier to manage. Caregivers frequently engaged in neglectful behaviors in response to physical aggression by their relative with dementia (32.1%). These findings indicate that while caregivers are aware of nonpharmalogical strategies to manage BSDs, and frequently use them as their first treatment, aggression and inappropriate behaviors may result in a breakdown of these strategies, where caregivers are more likely to revert to using medications as an alternative. These findings suggest emphasis should be placed on educating family caregivers on nonpharmalogical strategies that better manage aggressive behaviors. Work is needed to determine how frequently caregivers provide medication to manage behaviors and what influences their use of PIM.

